# Exploring determinants in deciding the optimal antimicrobial dose in patients undergoing CRRT: a mixed-methods study

**DOI:** 10.1128/aac.00238-25

**Published:** 2025-08-18

**Authors:** Tuğba Yanık Yalçın, Aysel Pehlivanlı, Fatma İrem Yeşiler, Helin Şahintürk, Özlem Azap, Hande Arslan, Bilgen Başgut, Pınar Zeyneloğlu

**Affiliations:** 1 Department of Infectious Diseases and Clinical Microbiology, Faculty of Medicine, Baskent University 37505, Ankara, Turkey; 2 Department of Pharmacology, Faculty of Pharmacy, Baskent University 37505, Ankara, Turkey; 3 Clinical Pharmacy and Drug Information Center, Baskent University Ankara Hospital 37505, Ankara, Turkey; 4 Department of Anesthesiology and Critical Care Unit, Faculty of Medicine, Baskent University 63994, Ankara, Turkey; University of California San Francisco, San Francisco, California, USA

**Keywords:** renal replacement therapies, drug dosage calculations, pharmaceutical arithmetic, qualitative evaluation, quantitative evaluation

## Abstract

Antimicrobial dosing in patients undergoing continuous renal replacement therapy (CRRT) is a clinical challenge. The study aimed to investigate the factors that influence appropriate antimicrobial doses in CRRT and to address gaps in current knowledge and practice. The mixed-method design involved two phases. For the quantitative phase, infectious disease, intensive care, and clinical pharmacy professionals completed a questionnaire assessing demographics and practice details, as well as their knowledge, attitudes, and practices on antibiotic dosing in CRRT. In the qualitative phase, online focus groups were conducted using a question schedule based on the Theoretical Domains Framework to explore challenges and expectations. A structured questionnaire was completed by 160 participants, most of whom were infectious disease specialists (61.3%) with over 10 years of experience (38.8%). Despite a high knowledge level of antimicrobial dosing during CRRT, the sieving coefficient was unclear at 74.4%. Although 96.3% reported adjusting doses, 78.8% lacked institutional guidelines, and 68.1% did not monitor drug levels. CRRT experience positively influenced knowledge and attitude scores, and different dosing practices were reported for meropenem, piperacillin-tazobactam, and vancomycin. Qualitative findings highlighted the need for standard guidelines and CRRT-specific training. Multidisciplinary collaboration and real-time monitoring of therapeutic drugs were emphasized. This study identifies key gaps in knowledge and practice regarding antimicrobial dosing in CRRT. Addressing these gaps requires targeted training programs, real-time drug monitoring, and the development of evidence-based dosing guidelines to enhance patient safety and antimicrobial efficacy. Future research should evaluate the impact of these interventions on clinical outcomes.

## INTRODUCTION

During the first week of an intensive care unit (ICU) stay, infection rates rise from 30% to 70%, with 70% receiving antibiotics (1). More than half of the patients with sepsis develop acute kidney injury (AKI) and require renal replacement therapy (RRT) and antibiotics simultaneously. In the early stages of sepsis, underdosing of antibiotics is a major concern due to altered pharmacokinetics, such as increased volume of distribution and enhanced clearance. These changes can lead to insufficient drug concentrations, reducing treatment effectiveness and potentially increasing the risk of resistance and poor clinical outcomes. Moreover, therapeutic antibiotic dosages should be determined quickly and accurately to enhance bacterial clearance and minimize adverse reactions (2, 3). For severe AKI, RRT is an effective treatment option. The goal of continuous renal replacement therapy (CRRT) is to mimic the kidney’s depurative function through a gradual and seamless extracorporeal blood purification process (4). Various factors can affect antibiotics pharmacokinetics (PK) and pharmacodynamics (PD) in patients undergoing CRRT, such as patient factors (e.g., obesity, malnutrition, and hypoalbuminemia), drug-related factors (e.g., lipophilicity, hydrophilicity, and molecular weight), and CRRT-related factors (e.g., filtration rate, filtration type, and volume). These factors hinder antimicrobials from reaching effective blood concentrations, leading to increased adverse reactions, antimicrobial resistance, and treatment failure (5).

There are inconsistencies among sources providing antimicrobial dosing recommendations during CRRT, and data on antibiotics dosages during CRRT are quite limited. Therefore, many complex learning-based models that are not supported by clinical data have frequently been proposed (6–9).

This mixed-method study aimed to identify determinants of optimal antimicrobial dosing in patients undergoing CRRT. The quantitative phase assessed healthcare professionals’ knowledge, attitudes, and practices (KAP) via a survey, while the qualitative phase explored contextual and behavioral factors through focus group discussions based on the Theoretical Domains Framework (TDF). This design enabled integration of measurable trends and deeper insights into clinical decision-making.

## MATERIALS AND METHODS

### Study design

**Fig 1:**
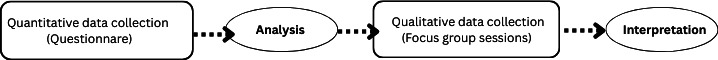
Mixed-method design schema.

An explanatory sequential mixed-method design was employed, in which the quantitative phase preceded the qualitative phase. The initial survey was conducted to identify patterns in KAP regarding antimicrobial dosing in CRRT. Subsequently, qualitative focus groups were conducted to explain and enrich the quantitative findings by exploring behavioral determinants through the TDF (Fig. 1). This approach allows for deeper insight into survey results and facilitates integration of numerical trends with contextual understanding (10). This study followed the Standards for Reporting Qualitative Research guideline, in line with EQUATOR Network recommendations.

### Eligibility criteria

Physicians (specializing in intensive care [IC] and infectious diseases [IDs]) and clinical pharmacists (CPs) involved in optimizing antimicrobial therapy for patients receiving CRRT participated in the survey. Among these participants, some expressed their willingness to take part in the subsequent focus group phase.

### Phase 1: quantitative study

#### Study instrument and development of survey

The survey was developed following best practices in instrument design, including a literature review, expert panel validation, item relevance rating on a 4-point scale, and calculation of the content validity index (CVI). The review covered studies and guidelines on antimicrobial dosing in CRRT published between 2010 and 2023, retrieved from PubMed, Scopus, and Web of Science using keywords such as “renal replacement therapy,” “CRRT,” and “antimicrobial dosing.” The structured survey form was first reviewed by an expert panel (including three ICs, two IDs, one pharmacologist, and one CP) to assess its clarity and validity. Also, the expert panel was asked to evaluate the items in the criterion in terms of intelligibility, serving the purpose, distinguishing, and cultural suitability by using the evaluation form and expressing their opinions by evaluating the measurement level of each item by 1–4 points. In the evaluation to be made regarding the intelligibility of each question, 1 point is “not appropriate”; 2 points are “somewhat appropriate, the item needs to be adjusted”; 3 points are “quite appropriate but minor changes are necessary”; and 4 points are “very appropriate” (11). The CVI is calculated using the percentage of agreement between the views. As a result of the experts’ answers, each item scoring 3 or 4 points above 80% is interpreted as a good CVI score (12). The suggested revisions were implemented. In order to determine a good fit for internal consistency, a Cronbach’s alpha result of more than 0.7 was considered acceptable. Additionally, each correct item was assigned 1 point based on a “yes” or “no” response.

As a result, a 26-question survey consisting of four sections was created (see Supplementary Material 1 at https://doi.org/10.6084/m9.figshare.29512151). The survey was structured as follows:

Section 1: comprised five questions that gathered general information such as age, gender, specialty (ID, IC, and CP), years of experience, institution, city, and the average number of monthly CRRT sessions.
Section 2: to assess the participants’ knowledge, six questions were asked using three response options: “yes,” “no,” and “not sure.”
Section 3: focused on the participants’ opinions regarding antibiotic dose adjustment in CRRT, with five questions.
Section 4: consisted of 12 questions related to clinical practice approaches in CRRT, including therapeutic drug monitoring (TDM), antibiotic dosing (meropenem, piperacillin-tazobactam, and vancomycin), infusion duration, loading, and maintenance doses.

#### Data collection

The survey, created using Google Forms, was distributed online to professionals in ID, IC, and CP. The survey link was sent via email through the mailing lists of relevant associations (The Turkish Clinical Microbiology and Infectious Diseases Society, Turkish Society of Intensive Care, and shared on social networks [WhatsApp]). The online survey was disseminated via email lists and professional messaging groups to approximately 400 healthcare professionals. Two reminders were sent 1 month apart to encourage participation. The survey was open for responses from April 2024 to June 2024. The sample size was determined based on prior studies on antimicrobial dosing in CRRT and the relatively small population of healthcare professionals involved in this field in Turkey. Considering the limited number of infectious disease specialists, intensivists, and clinical pharmacists working with CRRT patients, a target of 160 respondents was deemed adequate to capture a representative range of knowledge and practices.

#### Data analysis

Statistical analysis of the participant responses was carried out using Statistical Package for Social Sciences (version 22; Chicago, IL, USA). The frequencies and percentages were presented in tables using descriptive statistics. The mean difference in the knowledge, attitude, and practice scores was determined through analysis of variance and independent sample *t*-tests and presented in a table with means and standard deviations. A *P* value of <0.05 was considered statistically significant.

### Phase 2: qualitative study

#### Study instrument and data collection

The qualitative phase aimed to explore challenges, current practices, and expectations regarding antimicrobial dosing in CRRT. Questions (see Supplementary Material 2 at https://doi.org/10.6084/m9.figshare.29512151) were developed using the TDF to guide data collection and analysis, allowing systematic exploration of behavioral, contextual, and system-level factors. Three separate focus group sessions were conducted with 12 participants representing IDs, ICs, and CPs. Each group included four participants and was joined by a study team member from each specialty, ensuring a minimum of seven participants per session. Meetings were held online via Zoom and lasted between 30 and 60 minutes.

During the sessions, the questions were directed to the participants one by one, and their responses were recorded. The recordings were then transcribed verbatim, and their accuracy was verified by the research team through repeated listening and review of the transcripts to ensure reliability. Data collection took place in July 2024. Focus group sessions were preferred over individual interviews because they allowed participants to share and discuss their ideas or issues.

#### Data analysis

Transcripts were analyzed using framework analysis (13). Each focus group question was mapped to the predefined TDF domains. Qualitative data were coded according to the framework analysis, then the relevant themes were classified by linking them to the TDF domains. The themes obtained in line with this structure were analyzed in relation to each question. For coding, QSR International’s NVivo 12 Qualitative Data Analysis Software was used.

#### Data integration strategy

This study was conducted using an explanatory sequential mixed-method design. First, quantitative data were collected to assess the knowledge, attitudes, and practices of healthcare professionals in CRRT practices. Then, the qualitative data collection process was structured based on these findings. In particular, focus group questions were developed using the TDF in line with the trends and areas of uncertainty highlighted in the survey results. This approach allowed qualitative data to take on an explanatory and complementary role for quantitative findings. The integration process was mainly carried out in the analysis and interpretation phase. Qualitative findings reinforced some quantitative results (validation), deepened others (expansion), and presented different perspectives on certain issues (discrepancy). For example, while most participants stated that they made dose adjustments during CRRT (quantitative validation), focus group data showed that these practices were often implemented with difficulty due to unclear protocols and lack of communication (qualitative extension). This approach aligns with the principles suggested by Fetters and colleagues regarding data integration in mixed-method research (14).

## RESULTS

### Phase 1: quantitative study

#### Development and validation of survey

An expert panel evaluated each item according to its intelligibility, relevance, and differentiation. Each item was assessed according to a measurement level of 1 to 4. The CVI score was 0.90. The reliability of the survey was verified using Cronbach’s alpha and was found to be 0.8.

#### Demographics

A total of 160 participants completed the survey, with females being the majority (71.3%). Most (67.6%) participants were aged 20–40 years. The majority were infectious disease physicians (61.3%). Most of them had >10 years of professional experience (38.8%), and the number of CRRT patients they followed per month was ≤5 (63.1%) (Table 1).

**TABLE 1:** Characteristics of respondents (*n* = 160)

Variable	*n* (%)
Gender	
Female	114 (71.3)
Age (years)	
20–30	54 (33.8)
31–40	54 (33.8)
41–50	39 (24.4)
51–60	11 (6.9)
>60	2 (1.3)
Specialty	
Infectious diseases and clinical microbiology	98 (61.3)
Critical care	21 (13.1)
Anesthesiology	24 (15.0)
Clinical pharmacy	17 (10.6)
Experience (years)	
≤5	60 (37.5)
6–9	38 (23.8)
≥10	62 (38.8)
Institution type	
University hospital	79 (49.4)
Training and research hospital	49 (30.6)
Public hospital	22 (13.8)
Private hospital	7 (4.4)
Other	3 (1.8)
Monthly CRRT patients	
≤5 patients	101 (63.1)
6–9 patients	26 (16.3)
≥10 patients	33 (20.6)

#### Knowledge and attitude of health professionals about antimicrobial dosing during CRRT

While most of the participants answered correctly to four of the six questions (43.8%, 55.6%, 83.8%, and 38.8%, respectively), two questions were frequently answered incorrectly. Notably, 40% of respondents incorrectly believed that antimicrobials with a higher volume of distribution have a higher potential for removal by CRRT, whereas the correct answer was “no.” Furthermore, 74.4% of participants responded “I don’t know” to the question regarding sieving coefficient, despite the correct answer being “no,” indicating a major knowledge gap in this area. Among the participants, 36.9% agree that a higher dose should be given than the recommended dose in the guidelines. Most participants agreed that adjusting the dose in CRRT would reduce antimicrobial resistance (75.6%) and cost (73.8%). According to most health professionals (70.6%), pharmacists/clinical pharmacists should determine CRRT dosages (Table 2).

**TABLE 2:** Knowledge and attitude of respondents about antimicrobial dosing during CRRT^*a*^

Knowledge	*n* (%)	Attitude	*n* (%)
Antimicrobials with high molecular weight have a higher potential for removal by CRRT.		I think that antimicrobial drugs should be given in higher doses than the dose recommended by the guide I use in patients undergoing CRRT.	
Yes	48 (30.0)	Yes	59 (36.9)
No	70 (43.8)	No	77 (48.1)
Unknown	42 (26.3)	Unknown	24 (15.0)
Antimicrobials with a higher volume of distribution have a higher potential for removal by CRRT.		I think that antimicrobial drug dosage adjustment will prevent antibiotic resistance in the patient undergoing CRRT.	
Yes	64 (40.0)	Yes	121 (75.6)
No	61 (38.1)	No	21 (13.1)
Unknown	35 (21.9)	Unknown	18 (11.3)
Antimicrobials with high protein binding rates have a higher potential for removal by CRRT.		I think that adjusting the antimicrobial drug dosage in the patient undergoing CRRT will reduce the cost of drug use.	
Yes	35 (21.9)	Yes	118 (73.8)
No	89 (55.6)	No	22 (13.8)
Unknown	36 (22.5)	Unknown	20 (12.5)
The type of continuous renal replacement (hemofiltration, hemodialysis, or hemodiafiltration) does not alter the removal potential of the antimicrobial drug.		I think that adjusting the antimicrobial drug dosage in the patient undergoing CRRT will increase my workload.	
Yes	5 (3.1)	Yes	37 (23.1)
No	134 (83.8)	No	116 (72.5)
Unknown	21 (13.1)	Unknown	7 (4.4)
In intermittent hemodialysis, the removal potential of antimicrobials per unit time is higher than in CRRT.		I think antimicrobial drug doses should be monitored by the pharmacist/clinical pharmacist.	
Yes	57 (35.6)	Yes	113 (70.6)
No	62 (38.8)	No	39 (24.4)
Unknown	41 (25.6)	Unknown	8 (5.0)
In patients undergoing CRRT, the sieving coefficient or saturation coefficient of the drug being close to one means that its removal potential is low.			
Yes	16 (10.0)		
No	25 (15.6)		
Unknown	119 (74.4)		

^*a*^ Empty cells are used for formatting purposes only and do not represent missing or unreported data.

#### The practice of health professionals about antimicrobial dosing during CRRT

While almost all healthcare professionals (96.3%) stated that they adjusted antimicrobial doses in CRRT, many of them stated that there was no institution-specific guide (78.8%) and that they did not monitor antimicrobial levels (68.1%). The knowledge level of the participants who answered the first dose of meropenem as “30 minutes” (8.2 ± 1.6) was higher than the participants who answered, “I don’t know” (6.8 ± 2.1) (*P* = 0.003). The knowledge level of the participants who answered the first dose of piperacillin-tazobactam as “30 minutes” (7.8 ± 1.9) was higher than the participants who answered, “4 hours” (6.9 ± 2.2) and “I don’t know” (6.9 ± 1.9) (*P* = 0.04). While the attitude scores of the patients who applied the meropenem maintenance dose as "1 g three times daily" were higher (*P* < 0.001), the attitude scores of the participants who applied the piperacillin-tazobactam maintenance dose as “other” were higher (*P* = 0.007). The attitude score of those who answered the infusion time of the piperacillin-tazobactam maintenance dose as “30 minutes”(4.5 ± 1.9) was higher than the participants who answered “4 hours” (3.5 ± 1.9) (*P* = 0.031). In CRRT, the attitude scores of participants who did not apply (4.7 ± 1.9) a loading dose of vancomycin were higher than those who applied a loading dose (3.5 ± 1.7) (*P* = 0.002) (Table 3).

**TABLE 3:** The practice of respondents about antimicrobial dosing during CRRT^*a*^^,^^*b*^

Questions of practice	*n* (%)	Knowledge score		Attitude score	
		Mean ± SD	*P* value	Mean ± SD	*P* value
Can you adjust the antimicrobial dose in a patient undergoing CRRT? Yes No	154 (96.3) 6 (3.8)	7.4 ± 0.2 7.3 ± 0.8	0.633	3.9 ± 1.9 4.3 ± 1.9	0.818
Do you have a guideline with CRRT dose recommendations at your institution? Yes No	34 (21.3) 126 (78.8)	6.9 ± 2.3 7.5 ± 1.9	0.348	3.8 ± 1.9 3.9 ± 1.9	0.957
Can you monitor the serum levels of antimicrobials in your institution? Yes, in our institution. Yes, in another institution. No No, I find it unnecessary because the laboratory of the institution I work in gives late results.	41 (25.6) 7 (4.4) 109 (68.1) 3 (1.9)	7.9 ± 2.1 7.3 ± 1.2 7.1 ± 1.9 9.0 ± 1.0	0.072	3.9 ± 1.5 3.6 ± 2.7 3.9 ± 1.9 3.3 ± 1.1	0.892
Infusion time of the first dose of “meropenem” in your clinic in patients undergoing CRRT 30 minutes 3 hours Unknown Other	45 (28.1) 75 (46.9) 37 (23.1) 3 (1.9)	8.2 ± 1.6 7.1 ± 2.0 6.8 ± 2.1 6.7 ± 1.5	0.003*	3.7 ± 2.1 4.2 ± 1.8 3.6 ± 1.8 5.3 ± 1.1	0.155
Maintenance dose of "meropenem" applied in your clinic in patients undergoing CRRT 1 g three times daily 2 g three times daily Unknown Other	60 (37.5) 33 (20.6) 28 (17.5) 39 (24.4)	7.4 ± 2.0 7.4 ± 1.7 6.7 ± 1.8 7.7 ± 2.2	0.191	4.4 ± 1.9 2.7 ± 1.9 3.6 ± 1.7 4.5 ± 1.3	*P* < 0.001*
Infusion time of meropenem in patients undergoing CRRT 30 minutes 3 hours Unknown Other	28 (17.5) 95 (59.4) 35 (21.9) 2 (1.3)	7.8 ± 1.6 7.3 ± 2.1 7.1 ± 1.8 7.5 ± 2.1	0.576	4.1 ± 2.1 3.9 ± 1.9 3.6 ± 1.7 5.5 ± 2.1	0.448
Infusion time of the first dose of “piperacillin-tazobactam” in your clinic in patients undergoing CRRT 30 minutes 4 hours Unknown	75 (46.9) 38 (23.8) 47 (29.4)	7.8 ± 1.9 6.9 ± 2.2 6.9 ± 1.9	0.040*	4.1 ± 2.0 3.7 ± 1.7 3.7 ± 1.7	0.363
Maintenance dose of piperacillin-tazobactam applied in your clinic in patients undergoing CRRT 4.0 g/0.5 g three times daily 4.0 g/0.5 g four times daily Unknown Other	63 (39.4) 39 (24.4) 36 (22.5) 22 (13.8)	7.3 ± 2.1 7.6 ± 1.7 6.8 ± 1.9 7.9 ± 2.1	0.157	4.2 ± 2.1 3.2 ± 1.5^*a*^ 3.7 ± 1.8 4.8 ± 1.4^*a*^	0.007*
Infusion time of piperacillin-tazobactam in patients undergoing CRRT 30 minutes 4 hours Unknown Other	57 (35.5) 51 (31.9) 50 (31.3) 2 (1.3)	7.5 ± 1.9 7.5 ± 2.1 7.1 ± 2.0 6.5 ± 0.7	0.610	4.5 ± 1.9 3.5 ± 1.9 3.6 ± 1.7 4.0 ± 0.0	0.031*
Loading dose for “vancomycin” in your clinic in patients undergoing CRRT Yes No Unknown	76 (47.5) 45 (28.1) 39 (24.4)	7.7 ± 1.9 6.9 ± 2.0 7.2 ± 1.9	0.140	3.5 ± 1.7 4.7 ± 1.9 3.7 ± 1.9	0.002*
Maintenance dose of vancomycin administered in your clinic for patients undergoing CRRT 5 mg/kg twice a day 7.5–10.0 mg/kg twice a day 20–30 mg/kg twice a day Unknown Other	4 (2.5) 72 (45.0) 20 (12.5) 59 (36.9) 5 (3.1)	6.7 ± 1.7 7.6 ± 2.1 7.4 ± 1.6 7.1 ± 1.9 7.6 ± 3.0	0.683	4.5 ± 0.6 4.1 ± 1.9 3.6 ± 2.3 3.8 ± 1.7 4.8 ± 1.1	0.565
Infusion duration of vancomycin applied in our clinic in patients undergoing CRRT 1 hour 24 hours Unknown Other	91 (56.9) 9 (5.6) 52 (32.5) 8 (5.0)	7.6 ± 1.9 6.4 ± 3.1 7.2 ± 1.9 7.1 ± 2.3	0.336	4.1 ± 1.9 2.8 ± 1.5 3.7 ± 1.8 3.7 ± 1.7	0.155

^*a*^ * indicates *P* < 0.05*.*

^*b*^ CRRT: continuous renal replacement therapy; SD: standard deviation.

#### Comparison of knowledge, attitudes, and practice score

Participants with more than 10 years of experience (5.7 ± 2.2) had higher practice scores than others (≤5 years, 4.8 ± 2.1; 6–9 years, 4.6 ± 2.3) (*P* = 0.028). The number of CRRT patients followed in 1 month was higher in healthcare professionals with ≥10 practice scores (6.2 ± 1.9) than in those with practice scores of ≤5 (4.6 ± 2.1) (*P* < 0.001) (Table 4).

**TABLE 4:** Comparison of knowledge, attitudes, and practice scores^*a*^^,^^*b*^

Variables	Knowledge score		Attitude score		Practice score	
	Mean ± SD	*P* value	Mean ± SD	*P* value	Mean ± SD	*P* value
Gender		0.869		0.57		0.478
Female	7.3 ± 2.0		3.8 ± 1.9		5.1 ± 2.3	
Male	7.6 ± 1.9		4.1 ± 1.8		5.1 ± 2.1	
Age (years)		0.343		0.569		0.408
20–30	7.6 ± 1.8		3.8 ± 1.9		4.6 ± 2.2	
31–40	7.4 ± 2.1		4.2 ± 1.9		5.2 ± 2.3	
41–50	6.8 ± 2.0		3.7 ± 1.7		5.6 ± 2.2	
51–60	7.6 ± 2.1		3.5 ± 2.2		5.0 ± 2.0	
>60	7.5 ± 0.7		5.0 ± 0.0		5.5 ± 2.1	
Experience (years)		0.084		0.749		0.028*
≤5	7.8 ± 2.0		3.9 ± 1.8		4.8 ± 2.1	
6–9	7.0 ± 1.9		4.1 ± 1.9		4.6 ± 2.3	
≥10	7.1 ± 1.9		3.8 ± 1.9		5.7 ± 2.2	
Number of CRRT patients in a month		0.721		0.367		*P* < 0.001*
≤5	7.4 ± 1.9		4.1 ± 1.9		4.6 ± 2.1	
6–9	7.1 ± 2.3		3.6 ± 1.8		5.5 ± 2.4	
≥10	7.5 ± 1.8		3.7 ± 1.9		6.2 ± 1.9	

^*a*^ “*” indicates *P* < 0.05*.*

^*b*^ CRRT: continuous renal replacement therapy; SD: standard deviation.

### Phase 2: qualitative study

**Fig 2:**
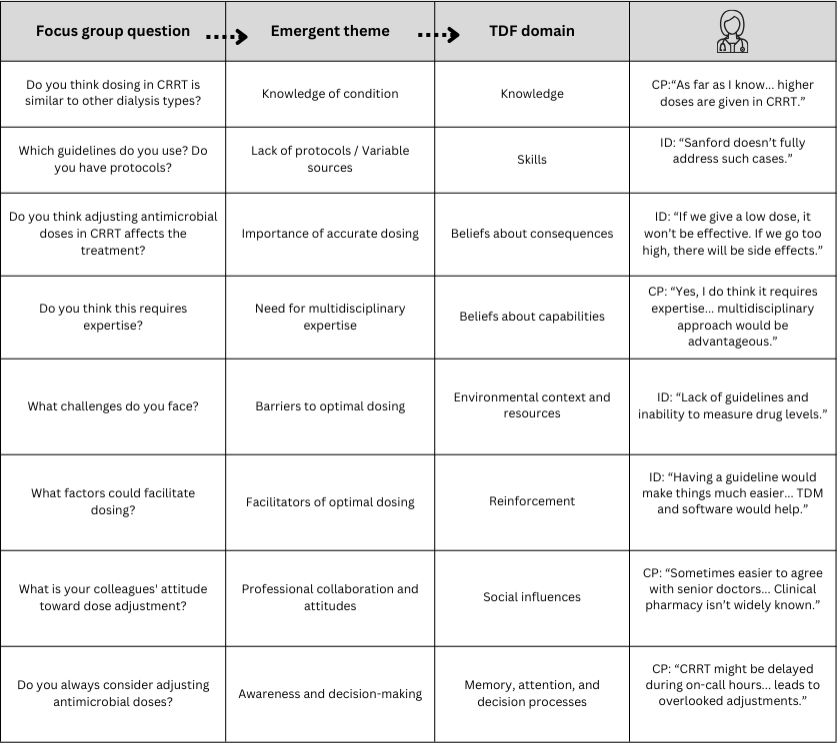
Details of focus group session themes and subthemes.

The themes derived from the focus group data were classified into eight main themes based on the TDF. Each theme was created based on predetermined questions and mapped to the relevant TDF domains. Figure 2 summarizes the interrelationship of the focus group questions, themes, and TDF domains.

### Theme 1: knowledge of condition

#### Do you think that antimicrobial dosing in CRRT is similar to the doses you apply in other types of dialysis?

All participants agreed that antibiotic dosing in CRRT differs from other types of dialysis and that higher doses are required.

ID: “Antibiotics can pass through those filters.”

IC: “Based on urine output. We actually consult with the infectious diseases department.”

CP: *“*As far as I know, in terms of both pharmacokinetics and application volumes, higher doses are given in CRRT*.*”

### Theme 2: skills

#### Which guidelines do you use when adjusting antimicrobial doses?

##### Do you have a protocol in your institution for the appropriate use of antimicrobials in CRRT?

All participants used the Sanford guide, but they expressed a lack of confidence in this source regarding antibiotic dosing in CRRT. Other sources used included UpToDate, Micromedex, and Lexicomp. While all IDs used Sanford, they conducted literature reviews for doses listed as insufficient data in Sanford. Some ICs adjusted doses themselves, while others deferred to infectious disease specialists. None of the centers had a protocol for antimicrobial dosing on CRRT.

ID: “For specific patients, for example, very septic ones with low albumin and a large volume of distribution. Sanford doesn’t fully address such cases.”

IC: “CRRT doses are highly variable. If the patient is in shock, we give the highest possible dose. We don’t know if we’re doing it right, but it’s like pressing all the buttons.”

CP: “UpToDate and Sanford meet our needs. If there’s no dosage information, we do a literature search, but we also consult peers.”

### Theme 3: optimism

#### Do you think adjusting antimicrobial doses in CRRT affects the treatment?

All participants agreed that insufficient dosing could lead to antimicrobial resistance and treatment failure, while excessive dosing could result in side effects.

ID: “If we give a low dose, it won’t be effective. If we go too high, there will be side effects. In the end, we might be giving too little or too much, which could manifest as treatment failure. Besides that, if we give too high a dose of something hepatotoxic, it could result in adverse effects.”

### Theme 4: beliefs about consequences

#### Do you think antimicrobial dosing in CRRT varies from patient to patient?

All participants agreed that antimicrobial dosing should vary based on the individual. They emphasized the importance of considering not only patient-specific factors (age, gender, and body fluid distribution) but also drug-specific factors and CRRT-related parameters.

IC: “Absolutely. Not only based on the patient but also on the drug. The pharmacokinetic and pharmacodynamic properties of the drug, the modality of CRRT, and the patient’s volume status all affect this. These factors can change the drug’s volume of distribution. Even the patient’s nutritional status could impact drugs that bind to proteins.*”*

### Theme 5: beliefs about capabilities

#### Do you think adjusting antimicrobial doses in a CRRT patient requires expertise?

##### What is your opinion on assigning this responsibility to a different healthcare team?

All participants agreed that adjusting antibiotic doses requires expertise, and they emphasized the need for training in this issue. All participants recommended a multidisciplinary approach, considering the complexity of CRRT and critical patient care, rather than assigning the responsibility to one person.

ID: “We're talking about CRRT, but especially for all critical patients, dose adjustment requires expertise. In my opinion, instead of assigning it to one person, it should be a collective effort. In Turkey, we can only measure the therapeutic levels of a few drugs. A clinical pharmacologist, or even a clinical pharmacist, would be utopic, though I’ve never worked with one.*”*

CP: “Yes, I do think it requires expertise, but I believe a multidisciplinary approach would be advantageous. A certification system or formal credentialing could also be considered.*”*

### Theme 6: environmental context and resources and reinforcement

#### What challenges do you face in adjusting appropriate antimicrobial doses in CRRT?

Participants ranked the challenges they face in dose adjustment in order of frequency:

- Lack of standardized guidelines and varying doses across different sources.
- Inability to measure therapeutic drug levels.
- Factors related to the CRRT modality.
- Patient-specific factors.
- Antibiotic-related factors.
- Awareness among healthcare teams.

#### What factors could facilitate appropriate antimicrobial dosing in CRRT?

Participants identified several facilitators for appropriate antimicrobial dosing:

- Having standardized guidelines.
- Therapeutic drug-level monitoring.
- Use of validated simulation software.
- Availability of non-filtered antibiotics by CRRT.

ID: “Having a guideline would make things much easier. We also need a well-coordinated team. Therapeutic drug monitoring, as well as validated simulation programs—there are such computer programs—would help. Additionally, it would be great if we had more antibiotics, especially ones that aren’t filtered by CRRT, which would simplify the selection process.*”*

### Theme 7: social influences

#### What is the general attitude of your colleagues regarding the adjustment of antimicrobial doses in CRRT?

ID: “We make decisions together with the intensivists. However, many times, they have made requests through official letters, saying things like, ‘We want to start antibiotics ourselves in the ICU without consulting infectious diseases. We put patients on ECMO, but we can’t choose the antibiotics.’ They want to decide about the antibiotic selection and dosing themselves. We face a serious problem with them on this issue.*”*

CP: “When you approach them with enough evidence-based information, people mostly accept your recommendations. That’s why, sometimes, it’s much easier to come to an agreement with senior doctors. Clinical pharmacy isn’t widely known or available in every department in Turkey.*”*

### Theme 8: memory, attention, decision processes, and behavioral regulation

#### Do you always consider adjusting antimicrobial doses in CRRT?

ID: “I suppose infectious disease specialists never overlook this.*”*

IC: “In patients using CRRT, adjusting the antibiotic dose before other pharmacological agents comes to mind*.”*

CP: “So, I don’t think that a situation like CRRT, which is relatively rare, would be overlooked. However, sometimes due to the intensity of the workload, we might face delays. It’s decided that it will be done, then canceled, and then started again. If it happens during on-call hours, we might not be there.”

## DISCUSSION

### Statement of key findings and integration of findings

This study identified critical factors affecting optimal antimicrobial dosing in CRRT. The quantitative findings revealed substantial variation in clinicians’ knowledge and practices, particularly around dose adjustment timing and TDM use. The qualitative phase provided deeper insight into barriers such as unclear professional roles, institutional protocol gaps, and perceived workload pressures. Integration of findings highlighted areas of both alignment and divergence between self-reported practices and contextual realities. Overall, the study underscored the need for clearer protocols, interdisciplinary collaboration, and behaviorally informed interventions. As attributed in most studies, participants agreed that, considering the disease and process, it is highly complex and difficult to standardize (15).

### Comparison with existing literature

Most participants believed that CRRT differs from other types of dialysis and that antibiotics should be administered at higher doses. The guidelines used by participants and the literature on antimicrobial dosing regimens in CRRT are conflicting. However, the dosages recommended in sources providing antibiotic dosing guidelines are inconsistent (16). The ability of CRRT to remove drugs can be affected by the drug’s characteristics. Molecular weight, protein binding, drug elimination, and volume of distribution (Vd) are considered the most important factors affecting PK parameters during CRRT (17). This inconsistency may be explained by the lack of essential PK/PD knowledge required to correctly interpret available studies and adjust doses accordingly, particularly in CRRT settings (18). In recognition of this gap, several international societies recommended routine use of TDM in critically ill patients as of 2020 to optimize antimicrobial exposure (19). Compared to hemodialysis, CRRT can remove high-molecular-weight drugs. Even some antimicrobials with high molecular weight can be effectively filtered under hemofiltration and hemodiafiltration modes. The free fraction of the drug is the portion that is removed, but when drugs are protein bound, it leads to the formation of large molecular compounds that are more difficult to remove. These factors affect drug clearance, leading to challenges in reaching the ideal blood concentration. On the other hand, studies conducted in this area are highly diverse. Some studies prioritize the hydrophilic or lipophilic nature of the drug, while others focus on concentration- or time-dependent antimicrobial effects, protein binding rates, or molecular size (20–24). In studies where TDM was performed, there are temporal differences in the collection of samples (25–27).

CRRT primarily significantly affects the clearance of drugs eliminated by the kidneys, but it can also increase the clearance of drugs eliminated by other organs. Drugs with a small Vd are typically hydrophilic and are usually eliminated by the kidneys. Meropenem is eliminated by the kidneys. A wide range of meropenem dosing regimens—from 0.25 g every 24 hours to 2 g every 8 hours—has been shown to be effective and is recommended for CRRT with various effluent flow rates (28, 29).

Extended or continuous infusion of beta-lactam antibiotics appears to be an effective method for enhancing clinical efficacy (30, 31). Among the participants, the general approach for meropenem was to administer it as a prolonged infusion, while prolonged infusion was less preferred for piperacillin-tazobactam therapy. About half of the participants administered the first dose of meropenem as a 3 hour infusion. However, subgroup analysis revealed that those who administered the first dose of meropenem over 30 minutes had a higher level of knowledge. In this critically ill group, it is crucial to rapidly reach the target therapeutic levels with loading doses of antibiotics. Several recent studies have demonstrated that prolonged or continuous infusion of β-lactam antibiotics improves the probability of achieving PK/PD targets, especially in critically ill patients with altered pharmacokinetics (19, 32). These strategies enhance percentage of time that the free (unbound) drug concentration is measurable during a dosing interval >minimum inhibitory concentration and may improve clinical outcomes, particularly in the setting of sepsis and CRRT, where standard dosing often leads to subtherapeutic exposure.

Most participants reported using piperacillin-tazobactam at a dose of 4.0 g/0.5 g every 8 hours, while approximately one-fourth preferred dosing every 6 hours. Approximately half of the participants administered the first infusion of piperacillin-tazobactam over 30 minutes, but for maintenance therapy, prolonged infusion was less preferred. In the survey study published by Tabah et al., it was also reported that piperacillin-tazobactam is mostly administered as a short-term infusion (33). Vancomycin is among the drugs for which TDM is recommended. Studies have shown that continuous infusion of vancomycin in patients undergoing CRRT reaches therapeutic levels more rapidly and maintains these levels within the target range (34). In the present study, we reported that prolonged infusion therapy with meropenem was more commonly preferred than piperacillin-tazobactam and vancomycin.

CRRT applications clear not only antibiotics, but also many other drugs (e.g., anticoagulants and antiepileptics) with critical therapeutic blood levels. During focus group sessions, it was noted that even if the dose adjustment of other drugs is overlooked, antibiotic dose adjustment is the first thing that comes to mind. Reasons for missing antimicrobial dose adjustments included starting CRRT during off-hours or uncertainty about whether CRRT would be performed during the day. Additionally, lack of collaboration among healthcare professionals was identified as a complicating factor.

While there was a strong need for a standard guideline, the concept of “one-size-fits-all” antibiotics in critically ill patients receiving CRRT might be feasible. All participants used the Sanford guide. The Sanford guide is an easily accessible resource, available as a mobile phone application. However, participants expressed a lack of confidence in the CRRT dosing recommendations provided by the Sanford guide. One of the most important factors contributing to patient variability—such as obese, cachectic, pregnant, or hydrocephalic patients—is altered volume of distribution. These groups of patients are needed to perform literature reviews and consult various sources.

This study found that many healthcare professionals prescribed antibiotics at higher doses than recommended by guidelines. They often consider these guidelines inadequate, particularly for critically ill patients whose pharmacokinetics may be altered and who require higher doses to achieve rapidly therapeutic effects (35). Antibiotic resistance further complicates this practice, as clinicians strive to maintain the effectiveness of their treatment against resistant organisms. In addition, healthcare professionals fear undertreatment in acute situations, which can lead to rapid clinical deterioration. The combination of these factors leads to higher antibiotic doses than recommended in clinical practice.

The strongest recommendation to facilitate antibiotic dose adjustment in CRRT was TDM. However, in clinical practice, TDM may not be feasible for every patient and every antibiotic. TDM is recommended in many scenarios, and studies have even shown it to be cost-effective (36). However, barriers need to be overcome. Even in centers where TDM is available, results are typically provided within 6–8 hours, leading to delayed antibiotic dose adjustments. In our study, 70% of the participants lacked the capability to perform TDM.

As another facilitator, participants proposed an innovative solution: validated simulation software. Although some examples of Bayesian forecasting and Monte Carlo simulation techniques exist, there is currently no software specifically for antimicrobial dose adjustment in CRRT (37–41). A software that could standardize antimicrobial dosing by inputting patient-specific and CRRT-related data would provide significant standardization. Another proposed solution, non-filtered antibiotics, might become possible with advancements in nanotechnology in the future.

Some physicians stated that they view collaboration with CPs as “utopian” due to several interconnected factors. First and foremost, a lack of familiarity with CPs’ roles and contributions has led to skepticism regarding the efficiency of such collaborations in Turkey. Furthermore, existing resources and staffing constraints may make the ideal of interdisciplinary teamwork difficult to achieve. Nevertheless, the integration of CPs into multidisciplinary teams—particularly in intensive care units—has been associated with improved clinical outcomes. CPs are specifically trained in pharmacokinetics and play a pivotal role in optimizing antimicrobial dosing in complex therapies such as CRRT. Evidence indicates that their involvement contributes to reduced mortality, shorter hospital stays, lower nephrotoxicity, and cost savings (42, 43). Communication and collaboration may be hindered by cultural barriers, including traditional hierarchies in healthcare. The integration of CPs into clinical decision-making processes may also be uncertain, especially in complex situations such as CRRT, which requires expertise from multiple disciplines. As a result of these challenges, collaborative care remains difficult to put into practice, despite its desirable characteristics. In this regard, strengthening and enhancing the role of CPs in developing nations such as Turkey may have a positive impact on antimicrobial drug use worldwide (44).

The integration of quantitative and qualitative data were done by considering the types of “fit” defined by Fetters et al. (14). A largely confirmatory relationship was observed between the data; for example, both survey and focus group data highlighted the lack of institutional guidance and the need for standardization in dosing. However, there were also explanatory findings; qualitative interviews revealed contextual barriers that could not be measured by the survey, such as operational difficulties in the dosing process and delays in shift hours. Partial mismatch was evident in the role of clinical pharmacists: survey participants supported this role, while focus groups indicated that this collaboration did not occur in practice and that there were structural barriers. This holistic approach was effective in providing the explanatory and depth intended in the mixed-method studies.

### Strengths and limitations

This study is among the first in Turkey to use an explanatory sequential mixed-method design to investigate antimicrobial dosing in CRRT. Integrating quantitative and qualitative data provided a more comprehensive understanding of practice-related behaviors, and the use of the TDF enhanced the rigor of the qualitative analysis.

Several limitations should be noted. The quantitative survey relied on self-reported data, introducing potential response and recall biases. While the sample size was appropriate for this specialized field, it may limit broader generalizability. The qualitative phase included a relatively small number of participants, although thematic saturation was achieved. Another limitation of the study is that a factor analysis was not conducted to statistically verify the theoretical structure of the survey. Although the content validity was provided by expert assessment and the internal consistency coefficient was at an acceptable level (*α* = 0.80), the structural validity of the knowledge, attitude, and practice dimensions was not tested statistically. This situation reveals the necessity of conducting structural validity analyses with methods such as exploratory or confirmatory factor analysis in future studies.

Finally, data integration occurred mainly during interpretation; future research could benefit from earlier-phase integration or intervention development.

### Further research

Further research is needed to address the identified gaps in knowledge regarding antimicrobial dosing in CRRT. Future studies could focus on planning and evaluating training programs to enhance healthcare professionals’ understanding of antimicrobial dosing, investigating the effectiveness of such training initiatives, and developing practical guidelines to standardize dosing practices.

### Conclusion

In critically ill patients, reaching therapeutic levels in the serum within the first hour is crucial, yet many confounding factors exist. In centers performing CRRT, intensivists and infectious disease specialists usually adjust the doses in our country. Clinical pharmacists are very interested and enthusiastic about this field, but their numbers are low. For many centers, it is still somewhat utopian. The number of centers performing TDM is quite limited, and there is a need to strengthen bioanalytical experts and laboratory support in this area. While individualizing antimicrobial dosing in CRRT seems challenging, there is an international need for a guideline in this field. The development of software could greatly facilitate standardization in this area.
